# Analysis of Blood Biochemistry and Pituitary-Gonadal Histology after Chronic Exposure to Bisphenol-A of Mice

**DOI:** 10.3390/ijerph192113894

**Published:** 2022-10-26

**Authors:** Ana M. Molina-López, Francisca Bujalance-Reyes, María Teresa Urbano, Antonio Lora-Benítez, Nahúm Ayala-Soldado, Rosario Moyano-Salvago

**Affiliations:** 1 Departamento Anatomía y Anatomía Patológica Comparadas y Toxicología, Unidad de Investigación Competitiva Zoonosis y Enfermedades Emergentes Desde la Perspectiva de Una Salud ENZOEM, Campus de Rabanales, Universidad de Córdoba, Edificio Darwin, 14071 Córdoba, Spain; 2 Departamento Anatomía y Anatomía Patológica Comparadas y Toxicología, Campus de Rabanales, Universidad de Córdoba, Edificio Darwin, 14071 Córdoba, Spain

**Keywords:** Bisphenol-A, endocrine disruptor, biochemical parameters, hypertriglyceridemia, hypercholesterolemia

## Abstract

Bisphenol-A is an emerging pollutant that is widespread in the environment, and to which live beings are continuously and inadvertently exposed. It is a substance with an endocrine-disrupting capacity, causing alterations in the reproductive, immunological, and neurological systems, among others, as well as metabolic alterations. Our study aimed to assess its clinical signs, and effects on the most relevant blood biochemical parameters, and to evaluate pituitary and gonadal histology after a chronic exposure of adult mice to different BPA doses (0.5, 2, 4, 50 and 100 µg/kg BW/day) through their drinking water. The biochemical results showed that a marked significant reduction (*p* < 0.05) was produced in the levels of serum glucose, hypoproteinaemia and hypoalbuminemia in the groups exposed to the highest doses, whereas in the group exposed to 50 µg/kg BW/day the glucose and total protein levels dropped, and the animals exposed to 100 µg/kg BW/day experienced a diminution in albumin levels. In the case of the group exposed to 50 µg/kg BW/day, however, hypertriglyceridemia and hypercholesterolemia were determined, and the blood parameters indicating kidney alterations such as urea and creatinine experienced a significant increase (*p* < 0.05) with respect to the controls. Regarding the pituitary and gonads, none of the animals exposed presented histological alterations at the doses tested, giving similar images to those of the control group. These results suggest that continuous exposure to low BPA doses could trigger an inhibition of hepatic gluconeogenesis, which would result in a hypoglycaemic state, together with an induction of the enzymes responsible for lipidic synthesis, a mechanism by which the increase in the lipid and serum cholesterol levels could be explained. Likewise, the decline in the protein and albumin levels would be indicative of a possible hepatic alteration, and the increase in urea and creatinine would point to a possible renal perturbation, derived from continuous exposure to this xenobiotic. Based on our results, it could be said that chronic exposure to low BPA doses would not produce any clinical signs or histological pituitary-gonadal effects, but it could cause modifications in some blood biochemical parameters, that could initially indicate a possible hepatic and renal effect.

## 1. Introduction

At present, mostly due to the increase in industrial activities, there has been an exponential rise in the presence of environmental pollutants that has become a threat to human and animal health. Bisphenol-A (2,2-bis(4-hydroxyphenyl) propane; BPA) is extensively used in the production of polycarbonate plastics and epoxy resins for a wide range of products used daily, such as food wrappings, drink cans, medical and electronic devices, thermal paper, etc. [1]; it is, therefore, a ubiquitous compound, to which living beings are continuously and inadvertently exposed. It has been estimated that its volume of manufacture will have exceeded 10 million tonnes in 2022 [2], which makes it one of the chemical products most produced worldwide. The potential sources of human exposure to it are basically foodstuffs (between 95 and 99%), which contain it via its migration from their packaging [3], although there are also other exposure pathways such as contact with the air, or the skin [4]. Although BPA is quickly metabolized in the organism, the presence of this compound in many objects used daily, and in the environment, leads to its continuous and unintentional exposure in living beings [5].

Bisphenol-A is regarded as being an emerging pollutant, resulting from, among other reasons, its migration from food containers and residues disposed of into the environment and that cause a serious environmental pollution problem, both in the landmass and aquatic spheres, mainly affecting all the exposed populations [6]. Given the complexity of natural systems, BPA may also present itself in combination with other environmental pollutants frequently found in the environment, and both its endocrine and mechanism actions could be enhanced or modified. Besides that, considering that some BPA metabolites have a higher oestrogenic potential than the compound itself [7], its effects on the environment could be underestimated, since wild species could be exposed to higher BPA concentrations in specific matrices such as leachates, fluvial and marine sediments, etc. [8]. In wild species, the evidence suggests that endocrine disruptors (ED) such as BPA could interfere with sexual development in a wide variety of species [9]. Another potential risk of BPA to some individuals of those species is the alteration in their reproductive and metabolic functions [10,11]. Numerous studies have focussed on the alterations occurring in free-living species, whose results could supply key information on the effects caused in humans, with the concept “One Health” acquiring special relevance in this context.

In view of its importance as a food and environmental pollutant, the action of BPA as a neuro-endocrine disruptor, and fundamentally as a disruptor of the pituitary–gonadal axis, has been amply studied [12,13,14,15,16]. Currently, special attention is being paid to the possible adverse effects related to the continuous exposure to low doses of BPA during a lifetime, since there is a great difference between reference doses (Tolerable Daily Intake, TDI) and the realistic levels of BPA exposure that have been determined in biomonitorization studies [17]. With regard to this aspect, the CLARITY-BPA report provides important information evidencing that exposure to low BPA doses triggers harmful effects. Indeed, the highest number of effects on reproduction were observed at doses 20,000 times lower than the current “safe” BPA dose for humans [18]. Currently, an evaluation made of a group of 148 bisphenols, among which was BPA, by the European Chemicals Agency (ECHA) and the Member States of the EU, has recommended restricting 30 of them, including BPA, due to their possible hormonal and toxic effects on reproduction. In view of the above, it is important to underline that one of the characteristics of EDCs is that they produce non-monotonic dose–response curves (NMDCR) in some dose ranges; a dose–response curve traces the intensity of an effect given in a range of doses examined. A NMDRC is characterized by a slope that changes its sign (trajectory) within the range of doses studied. Some curves are U-shaped, others are like an inverted U and, in others, the curve sign may change into multiple points throughout the dose range evaluated [19,20,21,22,23].

Bisphenol-A is an endocrine-disrupting substance, mimicking the action of oestradiol (E2) on binding with the receptors ER-αand ER-β, although with a lesser affinity than 17-β-oestradiol [24]. The latter acts on fertility, development, reproduction, the neurological and immunological systems, and homeostasis, both in human beings and animals [25,26,27]. Numerous epidemiological studies have demonstrated the correlation between exposure to BPA and the onset of chronic diseases such as obesity, type 2 diabetes and cardiovascular pathologies [28,29,30,31]. Likewise, some experiments have shown that exposure to BPA causes weight increase, an alteration in the levels of glucose in the blood and resistance to insulin, as well as the development of dyslipidaemias and changes in lipid metabolism [32,33,34]. Those studies showed positive associations between exposure to BPA and the development of metabolic diseases such as obesity and type 2 diabetes [35,36]. Similarly, many studies in animals reported that exposure to BPA resulted in an intolerance to glucose, resistance to insulin and alterations in the homeostasis of glucose [37,38]. However, the action mechanism of the BPA in the organisms exposed is not absolutely clear, although many works have demonstrated that BPA induces its effects by means of the generation of free radicals and oxidative stress [39,40]. Those studies have revealed that BPA exposure could change the levels of oxidative stress indicators, and that the differences in the latter might be related to target tissues in the animals, the BPA administration route, the dose and the duration of exposure. In this sense, exposure to both high and low BPA doses could affect the homeostasis of the oxidative system in rodents, despite the sensitivity of each indicator being able to differ and long-term exposure to BPA having the capacity to cause an increase in the oxidative substances that would involve harmful health problems.

This endocrine-alteration capacity of BPA and the metabolic effects associated with diverse health impairments have become of increasing concern to health authorities worldwide, with the European Authority of Food Safety (EFSA) reducing in 2015 the Tolerable Daily Intake (TDI) of 50 µg/kg BW/day to 4 µg/kg BW/day. At present, the EFSA is re-evaluating the risks of BPA in food and proposes to significantly reduce the TDI to 0.04 nanograms/kg BW/day. The draft of this document was published on 15 December 2021, and a period of consultation was initiated that ended in February 2022. It indicated that the lowering of the TDI resulted from the assessment of studies that emerged in the literature from 2013 to 2018, particularly those reporting the adverse effects of BPA on the immune system. By comparing the new TDI with estimates of consumer exposure to BPA in the diet, the EFSA concludes “those with both average and high exposure to BPA in all age groups exceed the new TDI, indicating health concerns”.

In in vivo studies on BPA, body weight data, hepatic and renal toxicity, organ weight changes and histology or blood biochemistry are considered to be criteria for evaluating the toxicity of BPA and, therefore, effect biomarkers could be useful for improving the assessment of risks of chemical products with a short half-life in the organism as is the case with BPA. The WHO and the Committee for Human Biomonitoring for Environmental Toxicants (IPCS-WHO, 1993, NRC, 2006), define effect biomarkers as” a biochemical, physiological, behavioural, or any other measurable type in an organism that, depending on their magnitude, can be recognized as being associated with an established or possible deterioration in health”. Thus, biochemical parameters could be used, with others, as indirect effect biomarkers of this environmental and food pollutant, and they could be useful for evaluating physical changes in free-living animals [11,41].

At present, most experimental studies are focused on short-term perinatal exposure to BPA, at doses above those established as being “safe” for humans during a short span of time, in the perinatal period, or during crucial stages of the experimental models’ lives. However, there are fewer works investigating the long-term effects of BPA at adult age. In this study. we therefore proposed to simulate a continuous and inadvertent exposure to BPA, in order to determine the effects of chronic exposure at ultra-low doses considered as being safe. Mice were exposed to BPA as from 8 weeks of age (the moment at which they are regarded as being sexually mature) for 52 weeks to investigate the effects on their blood biochemical parameters, as well as any possible pituitary and gonadal histopathological alterations, using a wide range of doses (0.5, 2, 4, 50 and 100 µg/kg BW/day) selected on the basis of recently published studies that include both estimated levels of exposure from the environment and from the diet [42].

## 2. Materials and Methods

### 2.1. Animals

Forty-eight (8-week-old) male and female mice (*Mus musculus*, C57BL/6jRj) were divided randomly into six groups (*n* = 8/group) (Table 1) and exposed as from 8 weeks of age for 52 weeks at different doses of BPA via their drinking water. The animals were housed individually in unventilated cages, measuring 45 × 25 × 15 cm with a bed of poplar shavings and environmental enrichment material. For feeding, a standard irradiated diet for mice in pellets (Altromin^®^ LAS QCdiet^®^ Rod14-H, Lage, Germany) was used. The drinking water consisted of filtered and autoclaved tap water. The mice were kept under constant photoperiod conditions (12 h light/dark cycle) and temperature (22–23 °C). The air changes in the room were constant (15–20 renewal/h) and the relative humidity of the housing room and experimental area was maintained at approximately 58% in a range of between 40 and 70%.

The in vivo experiment procedure was performed in the Experimentation Animals Service (SAEX) at Córdoba University (Spain). All the procedures were approved of by the Bioethics and Biosecurity Committee of the above university and authorized by the appropriate authority (number 26/06/2018/104). They were always carried out in compliance with the directives of the regulation in force (Royal Decree 53/2013, Directive 2010/63/EU).

### 2.2. Experiment Design

A control and five dose groups (0.5, 2, 4, 50 and 100 µg/kg BW/day) (*n* = 8/group) were established (Table 1). The animals were assigned to each group according to their initial mean weight (Supplementary Table S1). Exposure to BPA (Sigma Aldrich^®^, St. Luis, MO, USA) was performed orally by means of their drinking water. From this moment on, a period of 52 uninterrupted weeks of chronic exposure to BPA was started. Routine observations were made daily through the cage throughout the study to detect clinical signs, behaviour and general appearance, morbidity and mortality. Likewise, making this coincide with the consumption and weight controls of the mice, a more detailed clinical evaluation was carried out of all the animals once a week, following the check list shown in Table 2. Food and water intake and the body weights of all the animals were measured weekly; the results, expressed as mean values, are given in Supplementary Table S2.

### 2.3. Determination of Biochemical Parameters from Serum

After the exposure period had elapsed, for the sample extraction process, all the animals were anaesthetized with an inhalatory general anaesthetic Isoflurane (IsoVet^®^ Braun VetCare, Voorschoten, The Netherlands), a procedure from which the animals did not recover. Intra-cardiac blood samples were extracted and centrifuged at 3000× *g*, 10 m. The sera obtained were immediately analysed for the measurement of the following biochemical parameters: Glucose (GLUC), triglycerides (TG), total protein (TOT PROT), albumin (ALB), creatinine (CREAT), urea (UREA), alkaline phosphatase (ALP-DEA) and total cholesterol (TOT CHOL). The biochemical determinations in the serum were carried out using the Atom A-15 automatic analyser (Biosystems S.A., Barcelona, Spain), with kits from the same commercial company.

### 2.4. Gonads, Absolute and Relative Weights

Before sacrifice, the animals were weighed, and immediately afterwards their gonads were extracted for the determination and comparison of their relative weights between exposure groups.

### 2.5. Histopathological Analysis of Gonads and Pituitary Gland

Ovaries, testes and pituitary gland samples were obtained from all the animals and processed, fixing them in formaldehyde (Sigma*^®^* Aldrich, Boulder, CO, USA), buffered at 10% at room temperature, dehydrated in an ascending scale of ethanol and embedded in paraffin (Sigma*^®^* Aldrich, Boulder, CO, USA). The first sections of each block (4 µm) were stained with haematoxylin/eosin for their histopathological study. The histological preparations were processed with a high-resolution scanner (Leica APERIO AT2) and the images evaluated in dedicated software (APERIO and Slide Manager Version 12.4.0.8015), which took the images.

### 2.6. Statistical Analysis

The data were analysed using the Statistical Package for the Social Sciences (SPSS^®^) version 22.0. The ANOVA F test was employed to determine the significant differences between the mean values of each group in the parameter “Total Cholesterol”, and the Tukey test for the post-hoc analysis. For the rest of the parameters, the non-parametric statistical test was used, and the differences between groups were evaluated by the Mann–Whitney U-test. Significant differences between the treatment groups and the negative control were determined at *p* < 0.05.

## 3. Results

### 3.1. Clinical Evaluation

The animals did not present any visible clinical signs in any of the study groups throughout the exposure period. No animals died during the study.

### 3.2. Biochemical Parameters

#### 3.2.1. Glucose

**Figure 1:**
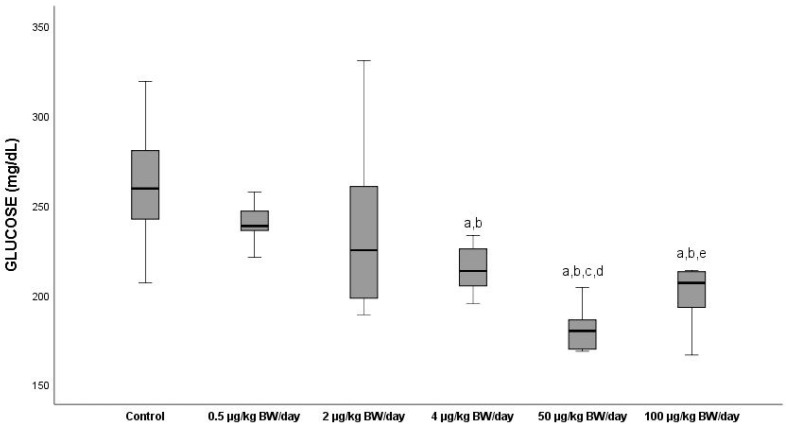
Means ± standard deviations of the serum levels of glucose (GLUC) expressed in mg/dL in the different exposure groups. ^a^ significant differences vs. control (G0) (*p* < 0.05); ^b^ significant differences vs. 0.5 µg/kg BW/day (G1); ^c^ significant differences vs. 2 µg/kg BW/day (G2); ^d^ significant differences vs. 4 µg/kg BW/day (G3); ^e^ significant differences vs. 50 µg/kg BW/day (G4).

The mean levels of glucose (GLUC) (Figure 1) diminished in all the exposure groups compared to the control group (G0) (mean value 261.19 mg/dL), with a significant decrease (*p* < 0.05) between the latter and the groups exposed to doses of over 2 µg/kg BW/day (G1), with the lowest decline (24.8%) being experienced by the group exposed to 50 µg/kg BW/day (G4) with respect to G0, as well as between the group exposed to 0.5 µg/kg BW/day (G1) and the rest of the groups exposed. There was an increase of 13.7% (*p* < 0.05) in the serum glucose levels of the animals exposed to the highest dose of BPA (G5: 100 µg/kg BW/day) with respect to those of group G4.

#### 3.2.2. Triglycerides

**Figure 2:**
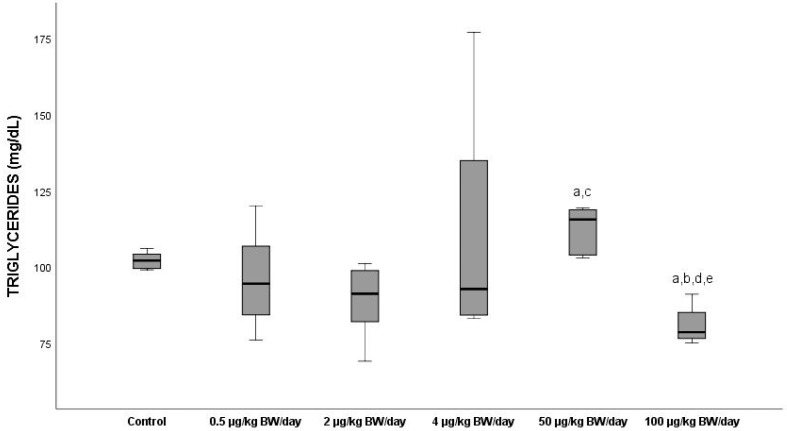
Means ± standard deviations of the serum levels of triglycerides (TG) expressed in mg/dL in the different exposure groups. ^a^ significant differences vs. control (G0) (*p* < 0.05); ^b^ significant differences vs. 0.5 µg/kg BW/day (G1); ^c^ significant differences vs. 2 µg/kg BW/day (G2); ^d^ significant differences vs. 4 µg/kg BW/day (G3); ^e^ significant differences vs. 50 µg/kg BW/day (G4).

The mean serum levels of triglycerides (TG) shown in Figure 2 in the groups exposed to the doses below 4 µg/kg BW/day decreased with respect to G0 (mean value of 101.66 mg/dL) (*p* > 0.05), whereas G4 presented hypertriglyceridemia (mean value: 114.39 mg/dL) experiencing an increase of 19.3% (*p* < 0.05) in TG levels compared to the G0. The group exposed to the highest dose (G5), however, displayed significantly lower mean values (*p* < 0.05) than the rest of the groups exposed, this being the sharpest decline (29.4%) with respect to G4.

#### 3.2.3. Total Proteins

**Figure 3:**
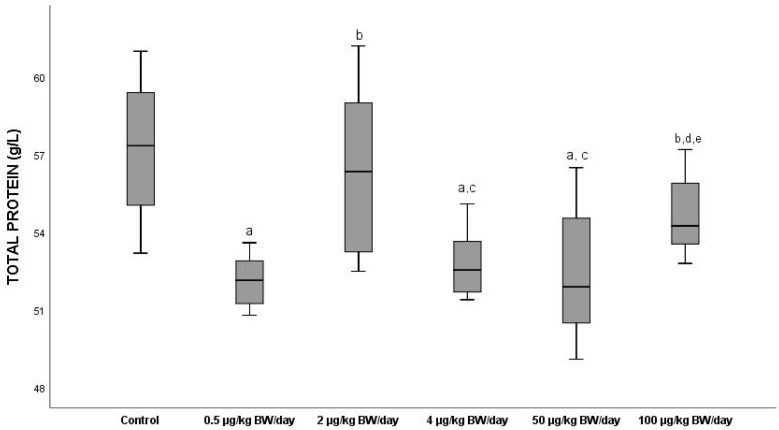
Means ± standard deviations of the serum levels of total proteins (TOT PROT) expressed in g/L in the different exposure groups. ^a^ significant differences vs. control (G0) (*p* < 0.05); ^b^ significant differences vs. 0.5 µg/kg BW/day (G1); ^c^ significant differences vs. 2 µg/kg BW/day (G2); ^d^ significant differences vs. 4 µg/kg BW/day (G3); ^e^ significant differences vs. 50 µg/kg BW/day (G4).

The mean levels of total proteins (TOT PROT) (Figure 3) diminished in all the groups with respect to the control that gave mean values of 57.3 g/L, these differences being significant (*p* < 0.05) between the control group and all the exposure groups except for the groups exposed to 2 µg/kg BW/day (G2) and 100 µg/kg BW/day (G5). Groups G1 and G4 underwent the most marked decreases in TOT PROT levels compared to G0 (8.9% and 8.4%, *p* < 0.05), respectively.

#### 3.2.4. Albumin

**Figure 4:**
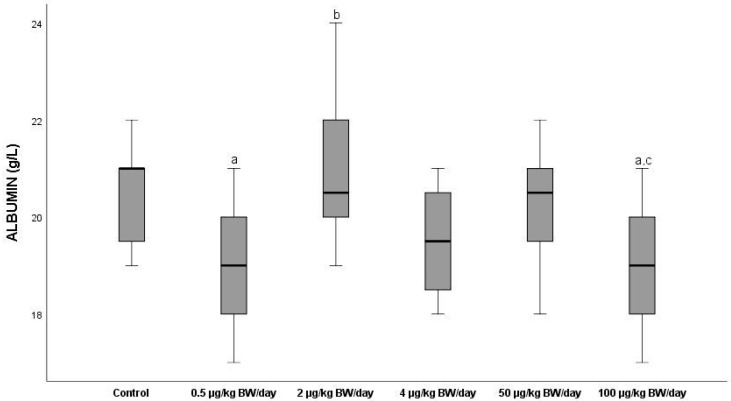
Means ± standard deviations of the serum levels of albumin (ALB) expressed in g/L in the different exposure groups. ^a^ significant differences vs. control (*p* < 0.05) (G0); ^b^ significant differences vs. 0.5 µg/kg BW/day (G1); ^c^ significant differences vs. 2 µg/kg BW/day (G2).

The mean levels of albumin (ALB) dropped by 7.3% (*p* < 0.05) with respect to the control (20.5 g/L) in the groups exposed, both in those at the lowest dose (0.5 µg/kg BW/day) and in those at the highest one (G1 and G5, respectively) (Figure 4). The animals exposed to 100 µg/kg BW/day (G5) underwent a diminution of 9.2% (*p* < 0.05) in the values of this biochemical parameter compared to G2, which gave the highest mean values (21 g/L).

#### 3.2.5. Creatinine

**Figure 5:**
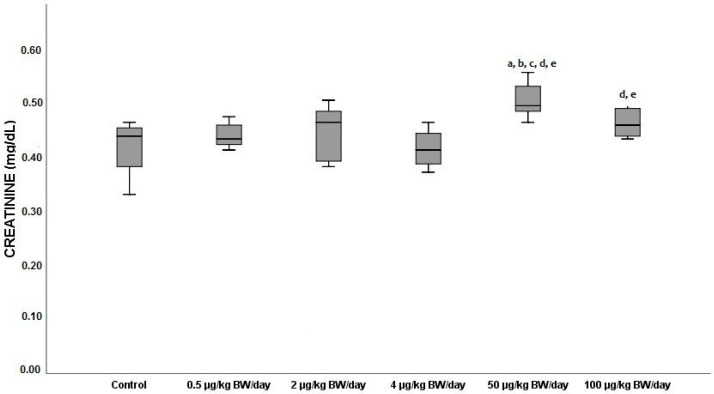
Means ± standard deviations of the serum levels of creatinine (CREAT) expressed in mg/dL, in the different exposure groups. ^a^ significant differences vs. control (G0) (*p* < 0.05); ^b^ significant differences vs. 0.5 µg/kg BW/day (G1); ^c^ significant differences vs. 2 µg/kg BW/day (G2), ^d^ significant differences vs. 4 µg/kg BW/day (G3); ^e^ significant differences vs. 50 µg/kg BW/day (G4).

With regard to the serum levels of creatinine (CREAT) displayed in Figure 5, the highest mean values were presented in the animals exposed to the dose of 50 µg/kg BW/day (0.46 mg/dL), at which those levels increased by 21% with respect to the control (mean value: 0.38 mg/dL) and 15%, 31% and 24% against the lower exposure doses (G1, G2 and G3, respectively, *p* < 0.05).

#### 3.2.6. Urea

**Figure 6:**
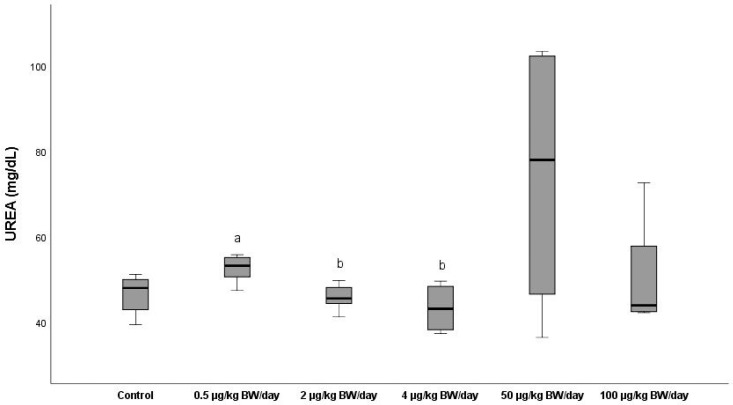
Means ± standard deviations of the serum levels of urea (UREA) expressed in mg/dL in the different exposure groups. ^a^ significant differences vs. control (G0) (*p* < 0.05); ^b^ significant differences vs. 0.5 µg/kg BW/day (G1).

The mean serum levels of urea (UREA) were significantly higher in G4 (74.2 mg/dL) than in the rest of the groups exposed and in the control group, (46.54 mg/dL), in which it rose by 59.4%, as can be observed in Figure 6. However, the group exposed to the highest BPA dose did not present any significant differences from the control group or from the rest of the groups exposed, except for G4, in which it experienced a decrease of 32%.

#### 3.2.7. Alkaline Phosphatase

**Figure 7:**
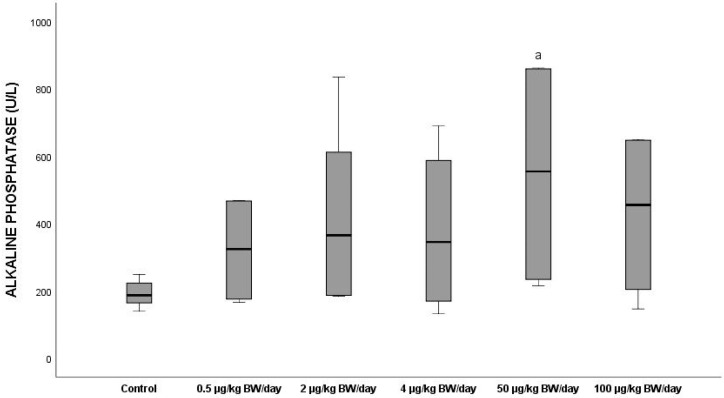
Means ± standard deviations of the serum levels of alkaline phosphatase (ALP-DEA) expressed in U/L in the different exposure groups. ^a^ significant differences vs. control (*p* < 0.05).

Exposure to BPA did not produce any notable effects on alkaline phosphatase (ALP-DEA) activity, although all the groups exposed gave higher mean ALP-DEA levels than the control group (192.63 U/L), Figure 7, the highest increase (184%, *p* < 0.05) being found in the group exposed to 50 µg/kg BW/day (mean value: 547 U/L)

#### 3.2.8. Total Cholesterol

**Figure 8:**
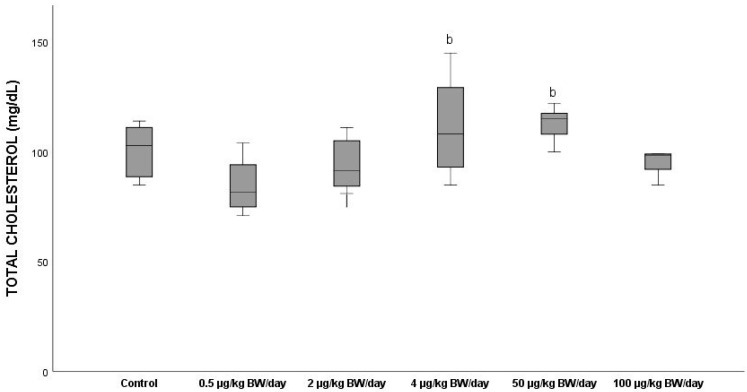
Means ± standard deviations of the serum levels of Total Cholesterol (TOT CHOL) expressed in mg/dL in the different exposure groups. ^b^ significant differences vs. 0.5 µg/kg BW/day (G1).

The total cholesterol (TOT CHOL) levels increased with respect to the control, showing mean values of 100.49 mg/dL in groups G3 and G4 (11 and 12%, respectively) (Figure 8). However, only significant differences (*p* < 0.05) were found between the group exposed to the lowest dose (0.5 µg/kg BW/day) (G1) and groups G3 and G4, which increased by 31 and 33.6%, respectively, compared to G1. Groups G1 and G2 showed lower mean values (84.50 mg/dL and 94.20 mg/dL, respectively), than the control group (100.49 mg/dL). Similarly, the animals exposed to 100 µg/kg BW/day (G5) gave lower cholesterol levels than those of G4 (112.88 mg/dL) and of the control group, showing a decrease of 15% and 5.1%, respectively.

### 3.3. Gonads, Absolute and Relative Weights

Gonad relative weight vs. total body weight recorded significant differences (*p* < 0.05) between most of the exposure groups. In the case of the males, the highest increase in this ratio compared to the control was found in G5, and for the females, all the groups experienced increases in those ratios with respect to the control group, those increases being significant ones in groups G2, G3, G4 and G5 (Table 3).

### 3.4. Histopathological Analysis of Hypophysis and Gonads

#### 3.4.1. Hypophysis

Macroscopically, no alterations in the morphology, colouring or size of the pituitary glands were observed in any of the animals.

**Figure 9:**
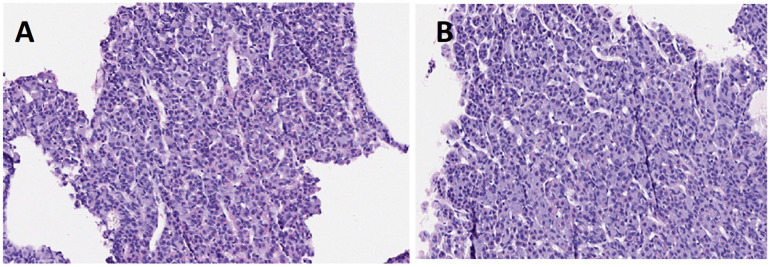
Histological sections of the hypophysis of the control group (**A**) and group G5 (**B**), (H-E 20×).

The histological studies did not exhibit any relevant alterations in any of the samples of animals treated when compared to the controls. In the section samples analysed, pituitary glandular tissue was examined, in which no degenerative changes or inflammatory response were noted in any of the samples (Figure 9).

#### 3.4.2. Ovary

Macroscopically, no lesions were observed in the morphology, colouring or size of the ovaries in any of the groups treated or in the control group.

**Figure 10:**
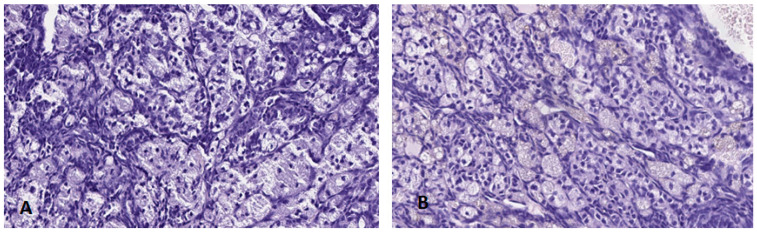
Histological sections of ovaries in female mice from the control group (**A**) and group 5 (**B**), (H-E 20×).

The ovarian tissue did not present any histological alteration and none of the samples of treated females analysed showed any change with respect to control group females (Figure 10), displaying a normal architecture with follicular structures with abundant interstitial lutein cells. Similarly, a well-differentiated peri-ovarian lymphoid tissue was noted.

#### 3.4.3. Testis

No notable morphological changes were observed macroscopically in the testes of the animals exposed to BPA with respect to the control groups, and there were no macroscopic changes in any of the cases of vascularization, hyperaemia or testicular congestion.

**Figure 11:**
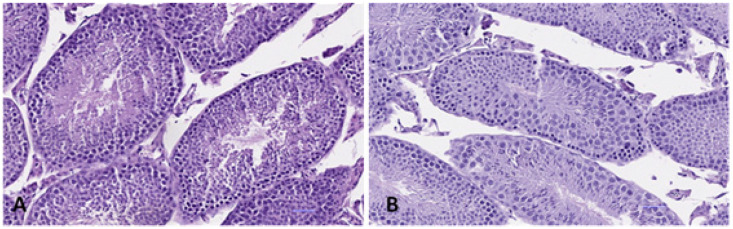
Histological sections of testis in male mice in the control group (**A**) 8.5× and group G5 (**B**), (H-E 14.5×).

The testicular tissue was observed as being histologically normal in all the animal samples, with no differences in the treated animals with respect to the control ones (Figure 11). It was a testicular tissue organized in tubules lined with a Germinal type and Sertoli type stratified epithelium over a fibroconnective basal lamina and with the luminal region containing abundant spermatozoids.

## 4. Discussion

The negative effects of BPA on human and animal health are a consequence of a chronic exposure to low environmental and food doses. Given their prevalence in the environment, the identification of BPA exposure/effect biomarkers could acquire a special relevance since they could be used to predict possible health effects. The exposure levels selected were those at which living beings could normally be exposed to BPA continuously and inadvertently as they are found as a pollutant in the environment and in foods. This study considered a wide variety of doses, including the current Tolerable Daily Intake (TDI), established by the EFSA at 4 µg/BW/day, and 50 µg/BW/day, the TDI in force up to 2015 and that is still valid in the USA according to the EPA and the American FDA. The dose of 0.5 µg/BW/day simulates the estimated environmental exposure [42] and numerous studies in animals have confirmed adverse effects in mice exposed to doses of 2 µg/BW/day. Finally, exposure to doses of 100 µg/BW/day has been demonstrated as having long-term harmful implications in the metabolism of some biochemical parameters such as glucose [43].

Regarding the levels of serum in glucose, the results showed a significant diminution (*p* < 0.05) in all the groups exposed, starting from 2 µg/kg BW/day compared to the control, this decrease being more marked (24.8%) in G4. The decline in those levels was progressive as from G2; however, a change was produced in the pathway of the curve in the serum levels of this parameter in G5, at which they began to increase with respect to the levels presented by the G4 animals. Coinciding with our results, Ji et al. (2020) [44] exposed rats during 35 days by oral gavage to low doses of BPA (1, 10, 50 and 250 µg/kg BW/day) and observed a drop in the levels of glucose serum in all the groups exposed that was significant at a dose of 250 µg/kg BW/day. These authors explained that BPA could upregulate the liver X receptor (LXR) in mice, and that hypoglycaemia would be caused by the LXR-regulated inhibition of the hepatic gluconeogenesis that would reduce glucose production and glycogen decomposition. For their part, Alonso-Magdalena et al. (2006) [45] reported that exposure of adult mice to a low BPA dose (10 mg/kg) triggered an increase in plasma insulin, and saw that a more prolonged exposure produced an increase in the insulin content in ß pancreatic cells in response to a stimulus of its oestrogen receptors with the mice developing chronic hyperinsulinism. This increase in chronic insulins could explain the low levels of glucose in blood at low levels of this endocrine disruptor. The mean levels of triglycerides in serum in groups G1 and G2 were reduced with respect to the control, whereas G4 presented hypertriglyceridemia, compared to the control group. Group five, however, gave significantly lower mean values (*p* < 0.05) than the rest of the groups exposed, this decrease being more marked (29.4%) than in G4. This non-monotonic behaviour is noted in Figure 2, in which a change in the slope of the curve is seen starting from the dose of 50 µg/kg BW/day, at which there is an increase in the levels of this parameter. However, latterly, there was a decline in G5, coinciding with the results obtained by authors such as Azevedo et al., 2020 [46]. Nevertheless, Haq et al. (2020) [47] reported a dose-dependent increase in this parameter after an intensive exposure at doses of 50, 500, 2500 and 5000 µg/kg BW/day compared to the control. Other authors found that exposure to BPA induced the gene expression of the enzymes intervening in the synthesis of lipids in rodents used as a biomodel [33,48]. Although some works have supplied evidence on the effect of exposure to EDCs on lipid metabolism [49], the molecular mechanism related to it is still unclear. Haq et al. (2020) [47] revealed that key metabolic enzymes such as hexokinase, acetyl-CoA carboxylase and squalene epoxide increased with exposure to BPA, which could justify the increase in the lipid profile observed in our study.

The total cholesterol levels displayed a similar non-monotonic behaviour to that presented by the serum triglyceride levels, in which there was a decrease in G1 with respect to the control, and, subsequently, a change in the dose–response curve trajectory, in which this parameter’s levels progressively increased up to G4 levels. Then, they declined again in G5, with the change in the curve trajectory (negative sign). In agreement with our results, Haq et al. (2020) [47] exposed mice for 16 weeks to low doses (50 and 500 μg/kg BW/day), showing an increased hepatic cholesterol content, suggesting that exposure to low BPA doses could trigger hepatic cholesterol synthesis, which would cause cholesterol accumulation and induce even further hepatic lipid levels, and alterations such as liver steatosis. A considerable number of experimental and epidemiological studies have demonstrated that exposure to BPA is related to lipid metabolic disorder and hepatic lipid accumulation [50]. In rodents, exposure to low BPA doses, both at perinatal and adult ages, could originate the accumulation of fat in the liver [50,51]. An excessive synthesis of cholesterol causes hypercholesterolemia and cholesterol accumulation in the liver that could produce hepatic lipid synthesis, and the onset of related diseases such as hepatic steatosis.

Our results regarding the mean levels of PT decreased in all the groups compared to the control, although the behaviour of the dose–response curve was typically non-monotonic, in which there was a notably significant decrease (*p* < 0.05) in G1 compared to the control, producing next a modification in the slope of the curve in G2, which was maintained up to G4, when it again changed its sign in the G5 animals. As for the mean albumin levels, these significantly diminished (*p* < 0.05) with respect to the control in groups G1 and G5 and displayed a similar non-monotonic dose–response curve to that of the PTs, except for G5, in which its trajectory was negative. These outcomes coincide with those obtained by authors such as Moon et al. (2012) [52], who also demonstrated this situation after the exposure of mice to doses of 50 µg/kg BW/day. However, in a study made by the consortium Clarity-BPA in rats, no significant differences were observed in PT levels in any of the groups with respect to the control at the continuous dose to which the animals were exposed at different concentrations of BPA (2.5, 25, 250, 2500 and 25,000 µg/kg BW/day) for two years.

Considering that the total protein levels in serum are a balance between their synthesis and their degradation, the hypoproteinaemia could be explained by taking into account that the administration of BPA would alter the integrity and functions of the liver, which is the principal organ implicated in biosynthesis of plasma proteins [53]. Besides that, there would be an increase in the urinary excretion of albumin since scientific evidence has confirmed that exposure to BPA causes proteinuria, associated with podocyte hypertrophy and an increase in the glomerular filtration rate. Thus, the reduction in the protein level could be indicative of hepatic damage possibly induced by BPA when the animals are exposed chronically to this xenoestrogen.

The hepatic damage suggested by the decline in TOT PRO and ALB could be seen better with a hepatic enzyme analysis such as ALP-DEA, which is released biologically into the bloodstream when the liver is damaged or when there is a biliary obstruction [54]. In our results, the ALP-DEA gave higher mean levels in all the groups exposed vs. the control group, with the highest increase in G4 (*p* < 0.05), coinciding with the results from authors such as Shi et al. (2021) [55], who, after exposing rats to low BPA doses, achieved a significant increase in liver dysfunction marker enzymes such as alkaline phosphatase. Conversely, authors such as Kazemi et al. (2017) [56], after exposing Wistar rats for 35 days to doses of 5, 25 and 125 µg/kg BW/day, obtained a significant decrease in ALP serum levels, also affirming that this short-term administration could cause liver damage. The greater hepatic enzyme activity might be explained by the alteration in the permeability of the hepatocyte membrane, induced by chronic exposure to BPA, in which case, the cell membrane would lose its integrity, originating a cell leak of these enzymes into the bloodstream. However, no increases were observed in the levels of hepatic enzymes such as ALP or AST in the NTP study (NTP Clarity Report, 2018), in any of the dose levels investigated (2.5, 25, 250, 2500 and 25,000 µg/kg BW/day) [18].

Regarding the serum levels in urea and creatinine, our results showed significant increases (*p* < 0.05) of these parameters in G4, compared to the control group. Both in the cases of the levels of urea and in those of creatinine, the behaviour of the dose–response was clearly non-monotonic. In the first case, the urea levels underwent an increase in G1 with respect to the control, and then again gradually diminished even in animals exposed to doses of 4 µg/kg BW/day. Next, a significant increase (*p* < 0.05) was seen in G4, which again decreased in the animals exposed to the highest dose, those levels of this parameter being like the control group. In the case of creatinine, the levels were the same as those of the control up to G3, and from then on, a turning point was reached that significantly increased (*p* < 0.05) the values of creatinine in G4 that were drastically reduced (*p* < 0.05) in the G5 animals. Authors such as Kobroob et al. (2018) [57] reported increases in these parameters in mice exposed to doses of 50, 100 and 150 mg/kg BW/day for 5 weeks, confirming evident kidney damage due to an increase in urea serum and creatinine, and the decline in creatinine clearance, together with the presence of proteinuria and dose-dependent glomerular lesions. Esplugas et al. (2018) [58] also observed kidney damage in mice exposed to low BPA doses (25 µg/kg BW/day) for 8 weeks, observing significant increases (*p* < 0.001) in the levels of serum urea in the group treated with BPA and the control group. Some authors such as Jiang et al. (2020) [59] hypothesized that BPA could cause functional damage in the kidney by triggering oxidative stress, inflammatory response and mitochondrial dysfunction after exposing rodents for 5 weeks to doses of 50 mg/kg BW/day. Our results could be indicative of the low BPA doses, after intensive exposure, causing alterations in blood parameters that could herald future kidney damage.

The relationship between BPA and some parameters such as adiposity and body weight exhibits a non-monotonic dose–response curve as has been shown in numerous studies [60,61]. This type of behaviour is probably due to its effects on the receptor’s kinetics and specificity, enabling it to act synergically with the endogenous oestrogen, which would explain why low BPA doses can generate effects on diverse biochemical parameters such as those related to lipid metabolism [62,63]. This characteristic behaviour pattern of EDCs is faced by the assumption on the part of the regulating organisms that tend to form linear relationships when setting up safe dose ranges, so that the establishment of the latter might not be suitable depending on the type of parameter it is aimed to evaluate. In this context, there are many studies showing that this linear behaviour does not occur after the valuation of certain effects. Additional effects also include a deficit in the sexual performance of male rats [64], and alterations in the hepatic expression of several genes involved in lipogenesis [50]. For its part, the non-monotonic response of BPA has also been demonstrated in different parameters analysed, including gene and protein expression, in hormone secretion, in lipid accumulation, in cholesterol biosynthesis and total biliary acids, etc. [65,66].

In the histological analysis of the tissues, we observed that, regarding the hypophysis, no relevant alterations in any of the samples of the animals treated vs. the controls were found. In agreement with our results, those from the central study of CLARITY-BPA after continuous exposure during two years of Sprague-Dawley rats at different doses of BPA (2.5–25,000 µg/kg BW/day), showed that the only lesion potentially related to BPA treatment was a significant increase in hyperplasia in the *pars distalis* of the hypophysis in the groups on a continuous dose of 25,000 μg BPA/kg BW/day, but only in the case of the males, although no neoplastic or non-neoplastic lesions were seen in the rest of the low-dose groups [67]. Therefore, coinciding with our results, no structural lesions in the hypophysis at low exposure doses were seen. Nor did our results determine any differences related to the sex of the animals, with none between males and females observed in the histopathological analysis, although the results given by Camacho et al. (2019) [67] ascertained differences at much higher doses than those tested in our study. With regard to pituitary damage, it is important to highlight a possible association between it and the exposure period of BPA. Thus, for the latter, during the development stage (prenatal and/or postnatal up to weaning), some studies of rats showed, in the histological investigation, an increase in hyperplasia in the *pars distalis* in females at doses of 2.5 and 25 µg/kg BW/day, and in males at 250 µg/kg BW/day. The effect of BPA on the pituitary gland could therefore be related to exposure during this crucial developmental stage [67]. Besides, it should be noted that our exposure period was initiated as from 8 weeks of life and during the adult age of the animals, with no lesions being observed microscopically in this organ.

Toxicity for both female and male reproduction and the exposure times (from development to weaning; from development to the adult age; during the growth phase; exposure in the adult age) are considered to be relevant valuation criteria when assessing BPA effects. The experiments in females showed that the effects on weight and ovarian histology derived from BPA exposure were less notable when exposure to this compound was affected during the adult age than during the developmental stages. As for the ovary weight, our results showed that there was an increase in their relative weight with respect to the control, and it was significantly (*p* < 0.05) higher in groups G2 and G3. In tune with our results, in the NTP Clarity Report 2018, a tendency to rise was observed in all the dose groups, except the group with the highest exposure dose (25,000 µg/kg/BW/day), in which the ovary weight was reduced. In another study with rats [68], approximately the same decline was observed in the ovary weight of F1 animals exposed to 0.5 y 50 μg/kg BW/day that had been exposed as from day 9 of gestation up to day 21 after birth (GD9-PND21). Also, Santamaría et al. (2016) [68] obtained ovary weight reduction at low doses with no dose-dependent effect. The results of these last two studies could indicate that the time of exposure during the crucial developmental stages could have a more decisive influence than the duration of exposure time on this parameter (ovary weight), it being important to underline that we did not find any studies on intensive exposure to BPA at the adult age, in which the relative weights of the gonads had been evaluated, which would have conferred a greater relevance to our results.

In the case of the females, the ovary tissues of the treated groups did not present any histological alteration (Figure 10). A normal architecture was exhibited in all the samples of treated females analysed, with no change with respect to the control ones. In line with our results, the continuous dose study of the CLARITY-BPA consortium found that histological effects of BPA in the ovary were limited to a significant rise in interstitial cell hypertrophy in the group on a continuous dose of 2500 μg BPA/kg BW/day, but no histological alterations below these dose levels were perceived. They were observed at doses 50 times higher than the highest dose administered in our study, so that it could be said that chronic exposure to low BPA doses (0.5, 2, 4, 50 and 100 μg BPA/kg BW/day) would not cause any histological alterations in the ovary.

In the case of male reproductive toxicity, some probable effects considered as being evaluation criteria include relative weight and testicular histology after the growth stage. Our results showed that the relative weight of male gonads diminished significantly (*p* < 0.05) in groups G2 and G3 with respect to the control group, and it subsequently increased again in exposure groups G4 and G5, displaying a typical non-monotonic curve behaviour. Our results differed from those of CLARITY-BPA, in which the testis weight did not reveal any significant differences in any of the dose levels tested, which covered ranges from 2.5 to 25,000 µg/Kg BW/day. Under the microscope, the testis tissue appeared to be histologically normal in all the animal samples, with no differences in the treated animals compared to the control ones (Figure 11). This contradicts what was observed by authors such as Tian et al. (2017) [69], who, after an oral BPA exposure (0, 100, 300 y 600 mg/kg BW/day) over 56 days in adult mice, reported disorders in spermatogenesis, lesions in the rough basal lamina of the seminiferous tubules and damage in the tight junctions between the Sertoli cells. Likewise, Jia et al. (2020) [70] observed histological damage in testicular tissues of mice exposed to 100 mg/kg BW/day, noting that BPA significantly decreased spermatogenic cells in the seminiferous tubules, and they observed a large vacuolization within the latter. In consonance with these last two authors, Sencar et al. (2021) [71] found serious degenerations such as testicular atrophy, spermatogenesis detention and interstitial oedema in the testicles of rats exposed during 28 days at concentrations of 100 and 200 mg/kg BW/day of BPA, demonstrating that only high exposure doses would originate the most serious effects in testicles. This might explain why, despite the exposure time of the animals in our study being 52 weeks, as the levels to which they were exposed were much lower than those indicated formerly (a thousand times higher than our maximum dose), no histological lesions were observed in the testes. Ullah et al. (2018a) [72] observed an increase in lipid peroxidation in the testicles of rats receiving a dose of 50,000 μg/kg BW/day for 28 days. In our study, the exposure levels were much lower than those previously evaluated by other authors; we did not observe any histological alteration in the treated animals with respect to the control, so that in the case of there being oxidative stress in the gonads, their organism could probably compensate for this by preventing the development of histological lesions.

## 5. Conclusions

Based on these results, it can be said that chronic exposure to low BPA doses (0.5, 2, 4, 50, 100 µg/kg BW/day), for 52 weeks, starting at the adult age, would apparently not have any visible clinical signs or histological effects on the hypophysis, ovary, or testis. On the contrary, the outcomes of the blood biochemical analysis showed that chronic exposure, even at low BPA doses, would cause hypoglycaemia, hypoproteinaemia and hypoalbuminemia, as well as an increase in the triglyceride and cholesterol in serum levels. All that might indicate effects on the liver and pancreas, in addition to the increase in blood parameters, revealing renal alterations such as urea and creatinine. Our results suggest that, through a blood biochemical analysis of living beings chronically exposed to BPA, together with other parameters, early detection of alterations occurring in different organs such as the liver, pancreas and kidneys could be achieved. This could end up by being of great importance in the monitoring of the effects of endocrine disruptors in free-living animals continuously and inadvertently exposed throughout their lives to this emerging pollutant. An understanding of the alterations in certain biochemical parameters in those free-living animals, plus an assessment of other parameters, could orientate epidemiological studies in humans, in which those changes could serve as indicators after exposure to this endocrine disruptor. The “One Health” concept would explain how the detrimental effects of BPA on different taxa in wildlife could supply key information on it in humans.

## Figures and Tables

**Table 1:** Group designation and target concentrations.

Target Concentration	Group Designation
Control	Group 0 (G0)
0.5 µg/kg BW/day	Group 1 (G1)
2 µg/kg BW/day	Group 2 (G2)
4 µg/kg BW/day	Group 3 (G3)
50 µg/kg BW/day	Group 4 (G4)
100 µg/kg BW/day	Group 5 (G5)

**Table 2:** Weekly Checklist inspection of animals (Adapted from Reporting clinical signs in laboratory animals from the FELASA Working Group Report, 2015).

Area Considered	Parts Considered	Detailed Observation and Reporting
**General**	Oversight	Nutritional and developmental status. Condition of the coat; Abnormal behaviour. Locomotor abnormalities
**Head**	Head (total)	Shape, size of parts and symmetry
	Nasal orifices	Opening, colour, moisture, discharges
	Mouth	Position, moisture, any wounds or deformities
	Eyes, eyelids	Position and close fit of the eyelids and eyeball. Transparency of cornea and any discolorations or discharge
	Ear (pinna, ear canal)	Shape of the pinna, inspection of the ear canal, any abnormal content or discharge
**Neck**	Part between head and shoulders	Shape and any wounds or masses
**Trunk**	Back	Position, symmetry, muscles
	Thorax	Respiratory movements
	Abdomen	Shape, tension, (respiratory) movements
	Pelvis	Mammary glands in this area, and inguinal lymph nodes.
**Limbs**	Front limbs	Wounds or deformities, proper use versus lameness
	Hindlimbs	Wounds or deformities, abnormal proper use versus lameness
**Tail**		Position, motility, tension (laxness or rigidity), any wounds or deformities.

**Table 3:** Body weight, ovary and testis weight and relative reproductive organs weight/body weight of male and female mice per study group.

		**Body Weight**	**Testis Weight**	**Ratio TES/BW**
**Dose Group**	**Sex**	**Mean ± SD**	**Mean ± SD**	**Mean ± SD**
**G0**	MALE	32.26 ±5.38	0.200 ± 0.01	0.625 ^b,c,d,e,f^ ± 0.06
**G1**	MALE	33.76 ± 3.98	0.225 ± 0.01	0.667 ^a,c,d,e^ ± 0.02
**G2**	MALE	36.43 ± 1.68	0.205 ± 0.03	0.562 ^a,b,c,e,f^ ± 0.01
**G3**	MALE	35.22 ± 5.64	0.205 ± 0.03	0.584 ^a,b,d,e,f^ ± 0.12
**G4**	MALE	29.48 ± 5.45	0.215 ± 0.01	0.730 ^a,b,c,d,f^ ± 0.05
**G5**	MALE	32.91 ± 0.82	0.215 ± 0.01	0.657 ^a,c,d,e,f^ ± 0.06
		**Body Weight**	**Ovary Weight**	**Ratio OV/BW**
**Dose Group**	**Sex**	**Mean ± SD**	**Mean ± SD**	**Mean ± SD**
**G0**	FEMALE	28.02 ± 0.77	0.025 ± 0.01	0.090 ^c,d,e,f^ ± 0.03
**G1**	FEMALE	30.59 ± 2.25	0.035 ± 0.02	0.113 ^c,d^ ± 0.06
**G2**	FEMALE	28.86 ± 2.44	0.075 ± 0.03	0.259 ^a,b,d,e,f^ ± 0.12
**G3**	FEMALE	28.47 ± 0.19	0.050 ± 0.01	0.175 ^a,b,c^ ± 0.04
**G4**	FEMALE	25.27 ± 1.35	0.040 ± 0.001	0.158 ^a,c^ ± 0.06
**G5**	FEMALE	29.95 ± 4.00	0.05 ± 0.001	0.150 ^a,b,c^ ± 0.03

Values are expressed as mean ± SD. ^a^ significant differences vs. G0, ^b^ significant differences vs. G1, ^c^ significant differences vs. G2, ^d^ significant differences vs. G3, ^e^ significant differences vs. G4, ^f^ significant differences vs. G5 (*p* < 0.05). The differences between groups for male and female were evaluated by ANOVA test or by Kruskal–Wallis test.

## Data Availability

Not applicable.

## Supplementary Materials

The following supporting information can be downloaded at: https://www.mdpi.com/article/10.3390/ijerph192113894/s1, Supplementary Table S1: Organization and target concentrations, Supplementary Table S2: Weights and Consumption Mean Values.
