# Supplementary material for: Analysis of Blood Biochemistry and Pituitary-Gonadal Histology after Chronic Exposure to Bisphenol-A of Mice
Source: Int J Environ Res Public Health. 2022 Oct 26;19(21):13894. doi: 10.3390/ijerph192113894 (PMC9659152; doi:10.3390/ijerph192113894)
Supplement: Supplementary file 1 [file ijerph-19-13894-s001.zip › ijerph-1894375-supplementary.pdf]

Supplementary Table S1. Organization and target concentrations

F0 INITIAL MEAN WEIGH (g) ± SD	DOSE GROUP
21.46 ± 3.25	**GROUP 0: CONTROL**
23.48 ± 3.79	**GROUP 1: 0.5 µg/Kg BW/day**
22.37 ±3.28	**GROUP 2: 2 µg/kg BW/day**
23.38 ± 3.66	**GROUP 3: 4 µg/kg BW/day**
21.42 ± 2.71	**GROUP 4: 50 µg/kg BW/day**
22.39 ± 3.48	**GROUP 5: 100 µg/kg BW/day**

Supplementary Table S2. Weights and Consumption Mean Values

DOSE GROUP	SEX	ANIMAL WEIGHT (g)		DAILY GAIN (g)		DAILY FOOD CONSUMPTION (g)		DAILY WATER CONSUMPTION (g)	
		Mean	SD	Mean	SD	Mean	SD	Mean	SD
Control	MALE	26.87	4.79	0.1	0.7	5.33	2.28	6.41	3.24
	FEMALE	30.34	4.5	0.11	0.64	4.27	0.7	4.05	0.99
0.5 µg/k/d	MALE	29.47	5.24	0.11	0.82	5.79	2.83	5.99	2.93
	FEMALE	32.13	3.26	0.1	0.62	4.44	0.72	4.43	1.41
2 µg/k/d	MALE	26.44	4.61	0.11	0.84	4.32	0.91	4.73	1.36
	FEMALE	33.5	5.03	0.13	0.78	4.28	0.88	3.69	0.77
4 µg/k/d	MALE	27.18	4.18	0.1	0.72	5.08	2	5.16	1.91
	FEMALE	34.95	4.13	0.12	0.76	4.49	0.82	3.98	0.77
50 µg/k/d	MALE	23.48	2.97	0.08	0.45	4.21	1.86	5.08	2.33
	FEMALE	29.84	2.78	0.09	0.59	4.3	0.98	4.76	1.69
100 µg/k/d	MALE	28.43	5.07	0.11	0.76	5.59	2.31	5.56	2.32
	FEMALE	32.56	3.48	0.11	0.66	4.63	1.84	4.34	1.4
